# DermO; an ontology for the description of dermatologic disease

**DOI:** 10.1186/s13326-016-0085-x

**Published:** 2016-06-13

**Authors:** Hannah M. Fisher, Robert Hoehndorf, Bruno S. Bazelato, Soheil S. Dadras, Lloyd E. King, Georgios V. Gkoutos, John P. Sundberg, Paul N. Schofield

**Affiliations:** Dept. of Physiology, Development and Neuroscience, University of Cambridge, Downing Street, Cambridge, CB2 3EG UK; Computational Bioscience Research Center, King Abdullah University of Science and Technology, 23955-6900 Thuwal, Kingdom of Saudi Arabia; Dept. of Computer Science, Llandinam Building, Aberystwyth University, Aberystwyth, Ceredigion SY23 3DB UK; Dept. Dermatology and Pathology, University of Connecticut Health Center, 263, Farmington Avenue, Farmington, CT 06030 USA; Dept. of Medicine, Div. Dermatology, Vanderbilt University, Nashville, Tennessee USA; College of Medical and Dental Sciences, Institute of Cancer and Genomic Sciences, Centre for Computational Biology, University of Birmingham, Birmingham, B15 2TT UK; Institute of Translational Medicine, University Hospitals Birmingham NHS Foundation Trust, Birmingham, B15 2TT UK; The Jackson Laboratory, 600, Main Street, Bar Harbor Maine, ME 04609-1500 USA

**Keywords:** Dermatology, Ontology, Disease, Dermatopathology

## Abstract

**Background:**

There have been repeated initiatives to produce standard nosologies and terminologies for cutaneous disease, some dedicated to the domain and some part of bigger terminologies such as ICD-10. Recently, formally structured terminologies, ontologies, have been widely developed in many areas of biomedical research. Primarily, these address the aim of providing comprehensive working terminologies for domains of knowledge, but because of the knowledge contained in the relationships between terms they can also be used computationally for many purposes.

**Results:**

We have developed an ontology of cutaneous disease, constructed manually by domain experts. With more than 3000 terms, DermO represents the most comprehensive formal dermatological disease terminology available. The disease entities are categorized in 20 upper level terms, which use a variety of features such as anatomical location, heritability, affected cell or tissue type, or etiology, as the features for classification, in line with professional practice and nosology in dermatology. Available in OBO flatfile and OWL 2 formats, it is integrated semantically with other ontologies and terminologies describing diseases and phenotypes. We demonstrate the application of DermO to text mining the biomedical literature and in the creation of a network describing the phenotypic relationships between cutaneous diseases.

**Conclusions:**

DermO is an ontology with broad coverage of the domain of dermatologic disease and we demonstrate here its utility for text mining and investigation of phenotypic relationships between dermatologic disorders. We envision that in the future it may be applied to the creation and mining of electronic health records, clinical training and basic research, as it supports automated inference and reasoning, and for the broader integration of skin disease information with that from other domains.

## Background

Estimation of the impact of skin disease on population morbidity and mortality has always been complicated by the nature of the terminologies used for recording disease incidence, prevalence and cause of death. However measured, it remains a highly significant social, economic and clinical burden. In the USA, collective prevalence estimates for skin disease are greater than those of obesity, hypertension and cancer [1]. There have been repeated initiatives to generate structured terminologies of sufficient granularity to accurately capture skin disease diagnoses, but the available tools, such as ICD-10, remain blunt instruments in the face of the pressing need for precision phenotyping, and are unsuitable for many types of computation-based research. We have, with the close involvement of domain experts in dermatology, pathology, and genetics, consequently created a new ontology, DermO, for cutaneous disease.

There have been several previous initiatives to devise lexicons or structured comprehensive terminologies for the description of cutaneous disorders, for example the Dermatologischer Diagnosenkatalog [2]. The most recent is the DermLex ontology [3, 4], created under the auspices of the American Academy of Dermatology, with the purpose of providing a definitive nomenclature for clinical dermatology [5]. This terminology was merged with the British Association of Dermatologist’s BAD Index and is mapped to International Classification of Disease (ICD9-CM) codes. However, maintenance of DermLex was discontinued in 2009. This lexicon covered sporadic and inherited cutaneous disorders and consisted of 6104 terms including a nosology, classic signs, therapeutic procedures, and anatomical distributions. To date DermLex has been the most comprehensive tool for capturing dermatological disease information. The Human Disease Ontology (DO) [6] also contains an integumentary branch of 234 terms, with skin and adnexal diseases classified as skin, hair, and nail disease. Likewise, the Human Phenotype Ontology (HPO) [7] contains a branch of skin and adnexal phenotypes with terms focused on phenotypic manifestations of cutaneous disorders. Skin diseases are largely out of the scope of HPO, which primarily focuses on phenotypes and does not aim to cover the breadth of cutaneous conditions we envisage. Similarly, the DO’s coverage and organization of the integumentary branch is limited and may not entirely capture the breadth and diversity of cutaneous conditions required for the purposes of patient stratification, population analysis, cross-species comparisons, database query expansion and automated reasoning.

The current revision of ICD, ICD-11, will contain the most radical revision of dermatology terms since 1948 and so far around 2000 terms have been assigned to the dermatology chapter (XII) with 20 major subdivisions [8]. ICD-11 is not currently finalized (June 2016) and uptake is expected to be gradual (for example the USA has only recently transitioned to ICD-10 for electronic health records as of 1.10.15) [9].

The other main resource for dermatological disease terms is SNOMED-CT. Whilst SNOMED contains more than 2000 terms related to cutaneous disease the coverage and structure of the terminology does not lend itself to detailed clinical description and having a relatively flat hierarchy does not permit the relationships between diseases to be used computationally in a useful way. (Discussed in [4]). An additional issue remains the licensing of SNOMED-CT by the International Health Terminology Standards Development Organization (IHTSDO) that precludes its free use in some countries.

The main applications for DermO are data capture and informatic analyses that only recently have become feasible using electronic health records, and semantic integration of disease information between species and across domains of knowledge; these analyses require a sufficiently rich structure, granularity, and coverage from the available terminologies. We have therefore created a new ontology from the scientific literature with the close involvement of domain experts in dermatology, pathology, and genetics, while explicitly maintaining interoperability with established ontologies and vocabularies in the biomedical domain, DermO is organized in a manner intuitive to dermatologists and dermatopathologists. The framework adopted for the development of DermO was based on that of the definitive clinical dermatology text, *Dermatology* by Bolognia et al. [10]. The specific aim is to include all of the currently accepted primary and secondary skin diseases, including those caused by systemic disorders, external insult, and the genodermatoses. DermO was developed with the intention of creating a tool applicable to patient care, clinical training and basic research, as well as to support automated inference and reasoning. It can be used for patient stratification, genotype/phenotype studies, and for the broader integration of skin disease information with that from other domains, such as model organism phenotypes and pharmacogenomics for translational science. DermO is freely available on https://github.com/dermatology-ontology/dermatology.

## Methods

### Ontology construction

DermO was constructed by domain experts using the framework of the most recent definitive text on Dermatology edited by Bolognia et al. [10]. The approach taken was to produce a classification familiar to dermatologists, as the envisaged uses of DermO include both patient diagnostic annotations by clinicians and mining of electronic health records. The formalization of patient information provides a data resource that can be expanded to integrate historical and newly generated information from both human and mouse dermatology, and genetic studies.

The structure adopted for DermO is familiar both for diagnostic support and patient data recording purposes. Because of the inclusion of systemic and inherited diseases, DermO also includes diseases that would normally be found in other branches of a disease ontology, such as Mendelian monogenic syndromes, and for completeness we therefore use a degree of polyhierarchy and include genetic diseases (the genodermatoses) as well as systemic diseases. For the same reasons the ICD-11 topic advisory group adopted a similar approach [11].

The ontology was manually constructed using OBO-Edit [12] and the OWL2 version prepared using Protégé [13]. Consistency was verified using the HermiT reasoner [14], which detected no inconsistencies and no unsatisfiable classes, mainly due to the absence of disjointness axioms in the ontology. The main ontology is available in both the OBO Flatfile format [15] and the Web Ontology Language (OWL) [16]. DermO is housed in a Github repository and is made available via Bioportal (permanent URL: http://purl.bioontology.org/ontology/DERMO) [17], Aber-OWL (permanent URL: http://aber-owl.net/ontology/DERMO) and on the project’s website https://github.com/dermatology-ontology/dermatology.

### Content, relations and mapping to other ontologies

**Fig. 1:**
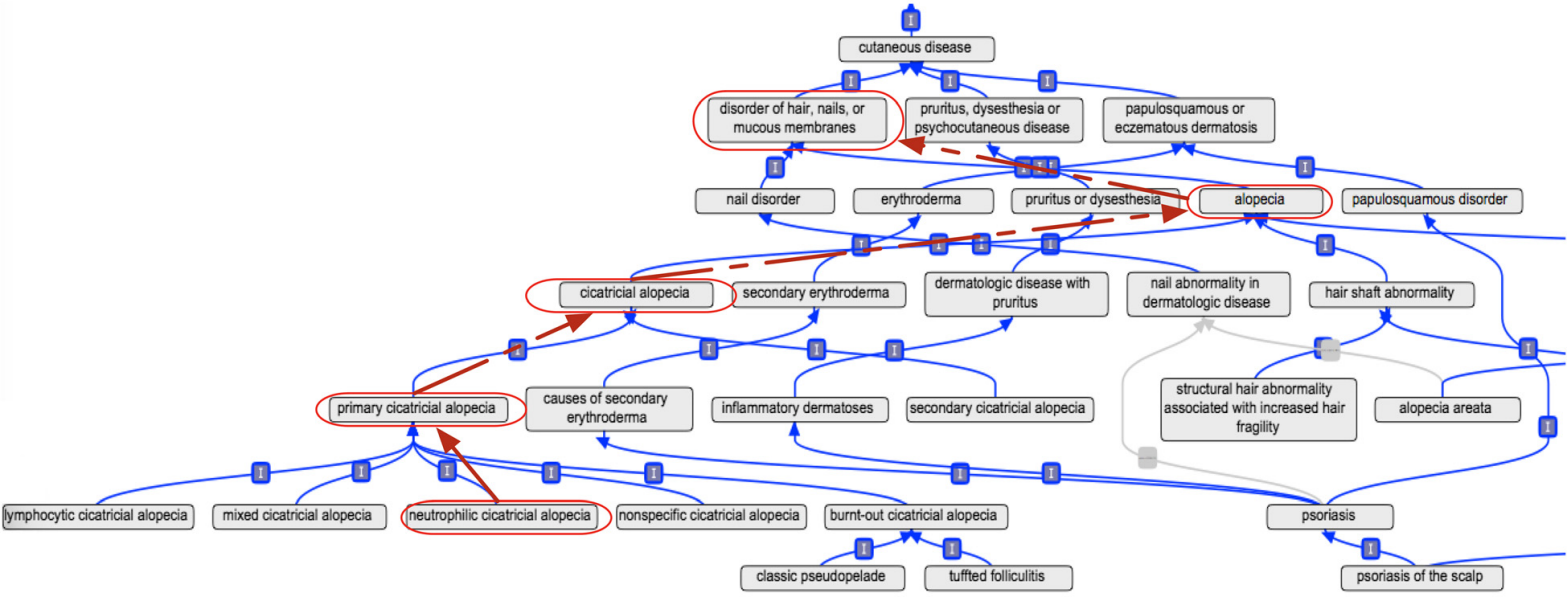
Part of the DermO ontology. The ontology classifies cutaneous diseases by etiology, anatomical location/cell type, and phenotype consistent with current clinical practice, and uses polyhierarchy (multiple parents) to maximize the types of knowledge captured by the ontology. The figure shows the path up the hierarchy for the classification of, neutrophilic cicatricial alopecia, with parent terms outlined in red and with a red arrow connecting parents and children. Multiple types of classification allow for multiple entry points and views of diseases. For example Psoriasis is a child of “papulosquamous disorder”, “inflammatory dermatoses”, and “causes of secondary erythroderma”. Most of the relations shown here are “is_a” – ie the child is a “type-of” its parent. Note that not all of the terms in this region of the ontology are shown here, for clarity

DermO was constructed as a simple ontology with limited polyhierarchy, and currently contains 3,425 classes (Fig. 1, Table 1). These are currently mapped to concepts in other major terminologies and provided with synonyms (Table 2). Synonyms were sourced from Bolognia et al. [10], DermnetNZ [18] and from expert curator knowledge. Class labels and synonyms were initially lexically mapped to concepts in UMLS [19], ICD-10, MPO [20], HPO [7], DO [6], OMIM (Online Mendelian Inheritance in Man) [21], SNOMED-CT [22], Medical Subject Headings (MeSH) and DermLex [4]. Only exact mappings were included and are provided as cross-references in the ontology. All were all manually verified. We also provide textual definitions for some of the diseases in DermO; creating further definitions is an ongoing process that has been greatly helped by participation of the Dermnet NZ [18].

**Table 1:** Upper level classes of the DermO ontology

Class	
Cutaneous disease	Adnexal disease
	Anogenital non-venereal disease
	Atrophy or disorder of dermal connective tissue
	Developmental anomaly
	Disorder caused by infections, infestations stings or bites
	Disorder due to physical agent
	Disorder of hair or nails
	Disorder of Langerhans cells or macrophages
	Disorder of neoplasm of the skin
	Disorders of subcutaneous fat
	Genodermatosis
	Metabolic or systemic disease
	Oral disorder
	Papulosquamous or eczematous dermatosis
	Pigmentary disorder
	Pruritis, dysaesthesia or psychocutaneous disorder
	Rheumatological disorder
	Urticaria, erythema or purpura
	Vascular disorder
	Vesiculobullous disease

The root class is cutaneous disease (DERMO:0000001) with 20 sibling child classes describing the head levels of the axes of classification and include etiology, anatomical location and inheritance, conforming to recognized criteria for classification of dermatological diseases from Bolognia et al. [10]

**Table 2:** Cross-ontology mapping of DermO classes

Ontology	Number of cross-referenced classes
Disease Ontology	1330
Human Phenotype Ontology	373
MeSH thesaurus	1509
UMLS	1398
ICD-10	537
DermLex	1054
SNOMED-CT	2337
Unreferenced classes	1773

Dermo classes were lexically mapped to a set of related ontologies and mappings subsequently manually checked. Only exact mappings were included but in many cases one DermO term could be mapped to classes in multiple ontologies with the same label or synonym. About half the classes in DermO have no match in the ontologies examined. Mappings are included in the ontology as cross references

The majority of relations used to structure DermO are *is*-*a* relations. Where appropriate, the *results*-*in* and *has*-*symptom* relations are also used to indicate where a disease develops as a sequel to another or where a disease is characterized by specific manifestations that might also occur in isolation. This latter relationship might also be viewed as that between a disease and phenotype as discussed below.

### Design considerations

Integration of the widest possible range of disease concepts into DermO brought with it challenges also seen in other phenotype and disease ontologies, such as DO and HPO which are dealt with in different ways, if indeed they are systematically addressed at all. The strategy we took was to allow multiple axes of classification, requiring polyhierarchy and resulting in a greatly increased richness. There are particular design considerations with the genodermatoses and etiologically predicated sub-types of disease. Our solutions and reasoning may be helpful to other developers of phenotypic and disease ontologies.

### Genodermatoses

The definition of a genodermatosis varies between terminologies [23] but broadly refers to inherited genetic skin conditions, often part of phenotypically diverse syndromes, and including mono-, polygenic, and chromosomal lesions. Probably the most comprehensive recent attempt to classify these is from Feramisco et al. [24, 25] who produced a database of genodermatoses derived from OMIM. They used manually curated phenotype terms to define the skin phenotypes of these genodermatoses to generate a phenotypic map of these diseases in order to discover novel relationships. We included most of the genodermatoses captured by Feramisco et al. into DermO, and also have added those considered by other studies, leading to a total of 537 genodermatoses in DermO. Furthermore, we have included subtypes of genodermatoses in phenotypic series and variants that did not receive their own OMIM identifier as separate manifestations rather than adopting the strategy of collapsing all the variants into a broad disease. For example, *hidradenitis suppurativa* (DOID: 2280) includes three subtypes of a phenotypic series (OMIM: 142690, OMIM: 613736, OMIM: 613737), all of which have a distinct phenotype and genetic etiology (*NCSTN*, *PSENN*, *and PSEN1* respectively). To facilitate studies of molecular mechanisms using DermO, such as classification in pathway analyses, patient population stratification, or identification of drugs for diseases in which each subtype may have different therapeutic indications, we separate the three sub-types of *hidradenitis suppurativa* into individual diseases. There are other cases where disease subtypes have no separate OMIM identifiers. For example, in the case of Darier-White disease (OMIM: 124200) we identify 11 subtypes. Only two are recognized in OMIM, “segmental” and “acral-hemorrhagic”, and neither have independent identifiers. No subtypes are listed for this disease in the genodermatosis database of Feramisco et al. We identify all of these as separate diseases in DermO so that their different etiology can be taken into account in data analysis.

### Etiologically predicated disease subtypes

Parallel classification of diseases by etiology allows for discrimination between the actions of different agents, which, although leading to the same outcome, may do so by different pathogenetic mechanisms – often termed “phenocopy”. In cases where this is well established, such as psoriasis [26], we have created subclasses of diseases based on the etiologic (causative or exacerbating) agent, such as virus, bacterium, or physical trauma, with the aim of being able to capture potentially important information for patient stratification and genotype/phenotype association. Such disease classes then have subclass relationships to classes that structure dermatological diseases based on their etiology. For example, fungal skin disease (DERMO:00002246) is a parent of “psoriasis triggered by fungal infection” (DERMO:0000004) [26], which has as its second parent “psoriasis” (DERMO:0000124). This enables identification of all the fungal skin diseases (including psoriasis triggered by fungal infection), or, using the other parent, identifying all the inflammatory dermatoses.

### Literature mining and network construction

Literature mining and construction of a phenotypic relatedness network for DermO classes was carried out as previously reported by us for the DO [27], and full methods may be found in this paper. In brief, the Aber-OWL: Pubmed infrastructure was used to semantically mine Medline abstracts. Aber-OWL: Pubmed (http://aber-owl.net/aber-owl/pubmed/) consists of an ontology repository, a reasoning infrastructure providing OWL-EL reasoning over the ontologies in the repository, a full text index of all Medline 2014 titles and abstracts as well as all Pubmed Central articles, and a search interface as described in Hoehndorf et al. [27]. Documents were dealt with as a title and abstract and filtered by presence of at least one term from a phenotype ontology (MP or HPO) and one term from DermO. The resulting indexed corpus consisted of 781,000 documents.

Statistically significant co-occurrences of disease and phenotype terms were identified using Normalized Pointwise Mutual Information (NPMI) [28] with the aim of associating HPO and MPO classes with individual concepts from DermO.

**Fig. 2:**
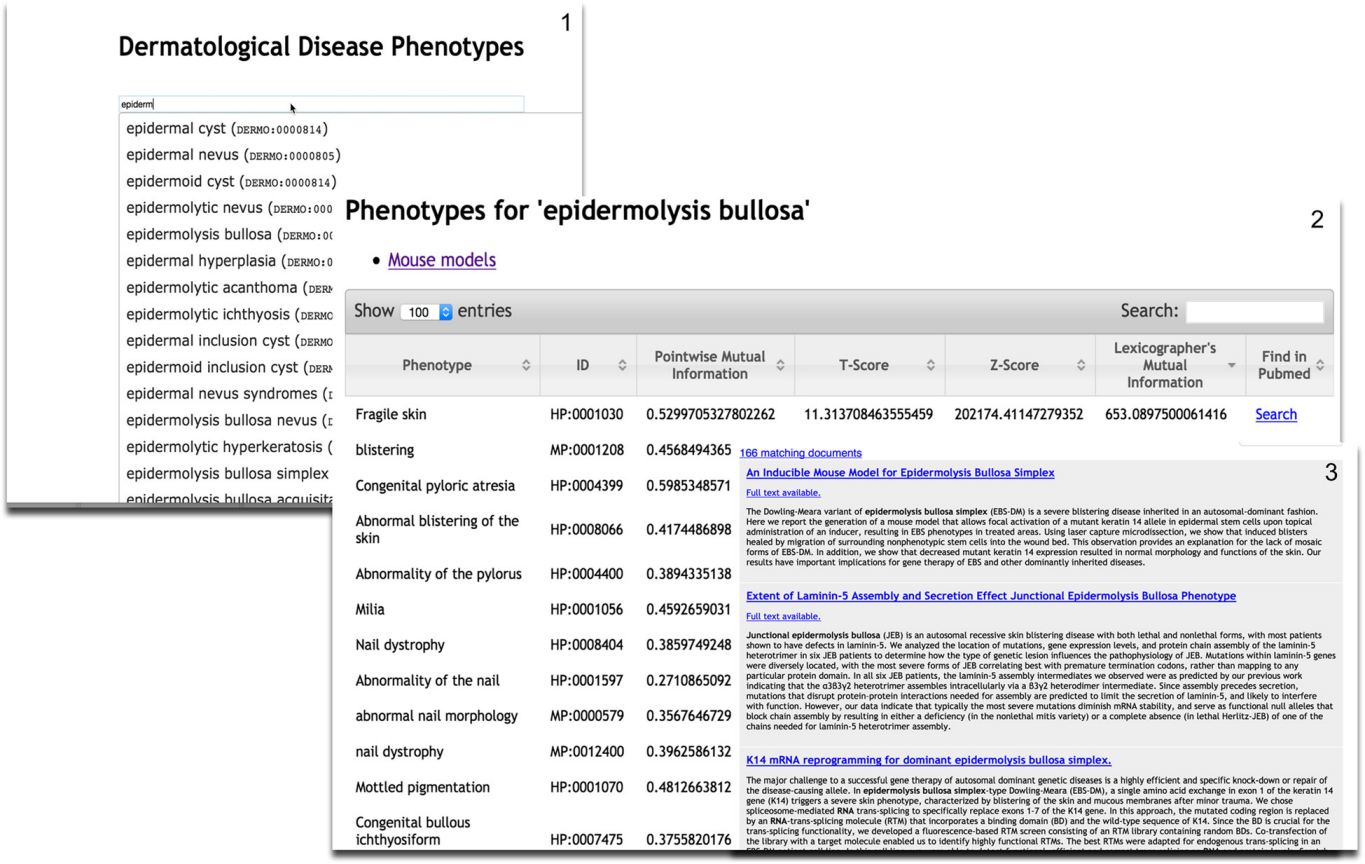
The Aber-Owl dermatological disease web page (http://aber-owl.net/aber-owl/dermophenotypes/) allows users to select a disease from the drop down box (view 1) and then retrieve a list of terms form the human or mammalian phenotype ontologies that are associated with it in the literature (view 2). The prioritization of these associations is determined by various metrics described in Hoehndorf et al, [27]. Users are able to reorder depending on the metric they wish to use. Each association can then be related to the Pubmed papers and abstracts that gave rise to it by clicking on the “search” link on the right hand side of the page (view 3)

These phenotypes can be freely accessed on http://aber-owl.net/aber-owl/dermophenotypes/ (Fig. 2). [29] Phenomenet is then used to establish the phenotypic relatedness of DermO classes in order to generate a network and visualised using a force directed layout using the Gephi graph visualization tool [30]. The algorithm for establishing relatedness is fully described in [27].

## Results and Discussion

### Scope and structure of DermO

Diseases of the skin and its adnexa fall mainly into three major categories which are well recognized by dermatologists; disease originating in the skin (sporadically or in response to external insults) with mainly cutaneous manifestations (I), diseases of the skin resulting from systemic disease such as metabolic or endocrine disorders (II), and heritable or genetic disease where skin manifestations are part of a syndrome (III) and may either arise independently of other aspects of the syndrome, or as a secondary consequence. The nature of cutaneous diseases, as with other anatomically classified diseases, include hyperplastic, dysplastic, neoplastic, and degenerative disorders for example, but many disease entities include manifestations of several of these pathological processes. This complexity consequently provides problems in how to logically classify the diseases.

DermO aims to capture knowledge about the domain of human cutaneous disorders in the form of a directed acyclic graph. It does not contain information about devices, procedures, diagnostic measurements or patient management. The ontological definition of diseases as dispositions rooted in physical disorders, realized through pathological processes, as proposed by Scheuermann et al. [31], is consistent with the criteria used for inclusion in DermO and all are children of the root term “cutaneous disease”. However the distinction between “phenotypes” and “diseases” is less clear; an issue with most attempts to capture the clinical domain. For example “atopic dermatitis” (DERMO:0000122) occurs sporadically and in isolation, and is regarded as a cutaneous disease, but also occurs as part of more complex syndromes such as mental retardation, obesity, mandibular prognathism with eye and skin anomalies [32] (MOMES syndrome) (OMIM: 606772) and consequently is treated as a disease in DO (DOID:3310 “*atopic dermatitis*”) and a phenotype in HPO (HP:0000964 “*atopic dermatitis*”). We feel that in such cases the distinction between “disease” and “phenotype” is largely dependent on context and have consequently included all that may be regarded as clinical disease entities, as opposed to clinical phenotype observations, as part of DermO, with cross-references to other ontologies where they also occur.

The disease entities in DermO are categorized in 20 upper level terms which use a variety of features such as anatomical location, heritability, affected cell or tissue type or etiology, as the features for classification, in line with professional practice and nosology in dermatology (Fig. 1, Table 1). The maximal depth is 11, and the majority of axioms used to structure DermO use *is*-*a* relations. 1,330 terms are common to DermO and DO and 373 are common to HPO, 152 to the Mammalian Phenotype Ontology (MPO), 1509 to the Medical Subject Headings (MeSH) thesaurus [33], 1398 to the Unified Medical Language System (UMLS), 537 to ICD-10, and 1054 to DermLex. 1203 classes in DermO map to 2337 classes in SNOMED-CT (RF2 release; USA, September 2015) (see Table 2). About half the classes could not be mapped to any of the above terminologies (1773), while many were mapped to more than one terminology, indicating significant redundancy of classes within the set of ontologies we used.

### Using DermO to mine the biomedical literature

There currently exists no high quality comprehensive phenotypic description of complex and common dermatological diseases. Using the approach we pioneered to harvest phenotypic descriptions of common disease from the scientific literature we have applied the same methodology using the coverage and resolution provided by DermO. The results of this analysis provide a rich resource of the associated phenotypes for dermatological diseases, including symptoms and co-morbidities. Using these descriptions to establish the phenotypic relatedness of the disease concepts in DermO we can generate a similarity network that provides insights into the mechanistic and pathobiological nature of these entities. It also a provides a novel and objective axis of classification, different to that traditionally used in dermatology, with the potential for providing novel insights into the pathobiology.

**Fig. 3:**
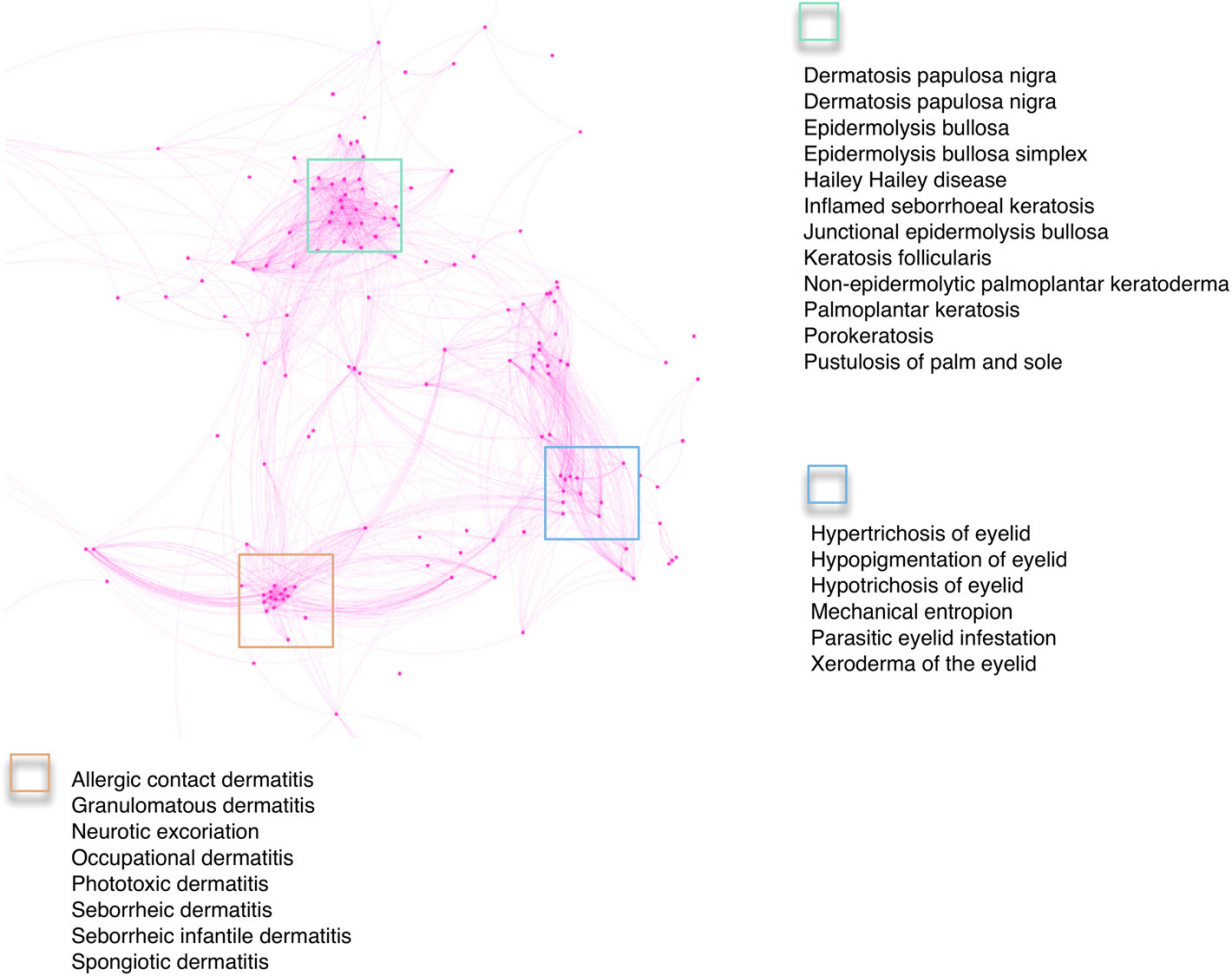
Clustering of skin diseases on the basis of phenotype similarity. The phenotypic profiles of each disease in DermO are related to each other through the phenotype ontologies and the strength of the association reflected in the presence and length of the lines connecting them. The algorithm for this is described in detail in Hoehndorf et al. [27]. This example shows close clustering of three types of skin disease, carried out automatically on the basis of the spectrum of text-mined phenotypes. This network is based on phenotype/disease associations established using NPMI [28] with phenotypic similarity determined using Phenomenet [36]

The area of the network shown in Fig. 3 contains several well-defined clusters of disease entity (in square frames): the dermatides; a cluster of epidermolytic dermatoses and keratoses; and a cluster of diseases of the eyelid, grouped by anatomy. Inclusion of some diseases within these clusters may at first sight be counter-intuitive, or at least counter-canonical. However when considering that the criteria for classification of diseases together in this case is purely phenotypic, they provide useful insights into the relationship between multiple disease entities. For example the inclusion of neurotic excoriation (DERMO:0000179) with the dermatitis cluster reflects the hair pulling and skin picking phenotypes associated with almost all types of dermatitis, shown here qualified by both etiology and pathological process. Clustering of several epidermolytic and keratotic diseases in the second cluster is interesting from the point of view of frequent co-occurrence in the same syndromic disease entities. These diseases are not usually included in the same group within standard terminologies, but clustering here reflects an underlying pathoetiological mechanism in some patients where disruption to epithelial junctions in the bullous diseases is often associated with hyperkeratosis and disruption of the dermal-epidermal junction. For example poro- and hyper- keratosis are seen together with bullous pathology in syndromic skin diseases such as the epidermolytic hyperkeratoses, and keratosis follicularis (Darier disease, (DERMO:0000064)) can present in bullous form [34]. Similarly palmoplantar keratoderma (palmoplantar keratosis), (DERMO:0000049) is also reported associated with blistering [35] reflecting an autoimmune origin of both phenotypes. The approach therefore enriches the pathobiological profile of well-established diseases and in some cases our mining of the literature highlights significant associations, sometimes in subsets of patients, that are often overlooked. An excellent example of this is the phenotypic description of Non-Herlitz type junctional epidermolysis bullosa (NHJEB), located in a neighbouring cluster (data from http://aber-owl.net/aber-owl/dermophenotypes/), where the text-mined phenotypes indicate a link with nail dystrophy. The *Lamc2* mouse, considered to be a model of NHJEB, does not show the nail phenotype flagged by our mining of the literature, which is relatively rare in humans, but introduction of a variant in a second locus, *Col17*α*1*, modified the disease to include the nail phenotype and model the subclass of human disease more accurately. This approach is now being followed for other dermatological diseases in the search for modifiers of canonical conditions using model organisms [Sproule TJ, Sundberg JP, Low BE, Silva KA, Reyon D, Joung JK, Wiles MV, Roopenian DC. TALEN-induced nested deletions and sequence replacements of collagen 17a1 reveal the functional basis of a genetic modifier of junctional epidermolysis bullosa in mice. PLos Genetics (submitted)].

The text-mined phenotypes are now integrated into Phenomenet [36] which allows for exploration of existing mouse models using the richer phenotypic descriptions of the skin diseases we provide. This supports disease gene discovery, where the gene (s) underlying a disease in humans is (are) unknown, and translational research. Such descriptions have not previously been available for common and complex skin disease.

## Conclusions

DermO was conceived as a formal ontology and structured terminology to describe human cutaneous diseases. DermO can be used as a tool in patient care, clinical training and basic research. The provision of disease relationships and groupings which have pathobiological and etiological significance based on phenotype makes DermO valuable for using automated inference and reasoning as well as in data mining applications, but we envisage wider applicability, from precision phenotyping of patients, through differential diagnosis, to the discovery of new physiological and pathophysiological pathways and integration of human and model organism phenotype data.

DermO can be used to extract data from patient electronic health records using text mining, or to translate existing variable-granularity coding such as ICD-10 to allow capture and standardization of patient/disease annotations. For example ICD-10 has 19 sub- types of bullous disorder including *Not Otherwise Specified* (NOS), whereas, excluding the bullous diseases of the newborn, DermO contains 84. Using the structure of the ontology patients may be grouped or split at different levels of granularity which will enable the fine-tuning of both Genome Wide Association Studies (GWAS) and Phenome Wide Association Studies (PheWAS) [37, 38] analysis. This will facilitate both the discovery of new genetic variation underlying skin diseases and the establishment of unexpected skin and other phenotypes associated with established genetic variants in and around genes identified as important through hypothesis-driven research. An additional advantage of this approach is the potential to identify other variation-associated predisposed phenotypes which might not have been noted previously, and which may shed light on the pathobiology of the disease through the introduction of novel and objective axes of classification by phenotype. In the future, we envision integrating DermO with the integumentary disease branch of the DO so that the deep and broad coverage of dermatological diseases we provide in DermO can be used in conjunction with other diseases within the DO. DermO, as with other open ontologies, is owned by the community that uses it and so we encourage users to participate in its development and improvement through the project website.

## Abbreviations

DermO: dermatology ontology
DO: human disease ontology
GWAS: genome-wide association studies
HPO: human phenotype ontology
ICD-9, 10, 11: international classification of disease versions 9, 10, and 11
MeSH: medical subject headings
MPO: mammalian phenotype ontology
NOS: not otherwise specified
OBO: open biomedical ontologies
OMIM: online mendelian inheritance in man
OWL: web ontology language
PheWAS: phenome-wide association studies
PNS: Paul N. Schofield
UMLS: unified medical language system
